# Diagnostic Value of High-Sensitivity Troponin T for Subclinical Left Ventricular Systolic Dysfunction in Patients with Sepsis

**DOI:** 10.1155/2021/8897738

**Published:** 2021-04-23

**Authors:** Pham Dang Hai, Nguyen Thanh Binh, Nguyen Hong Tot, Ha Manh Hung, Le Thi Viet Hoa, Nguyen Viet Quang Hien, Pham Nguyen Son

**Affiliations:** ^**1**^ Intensive Care Unit, 108 Military Central Hospital, Hanoi, Vietnam; ^2^Intensive Care Unit, Tam Anh Hospital, Hanoi, Vietnam; ^3^Department of Anesthesiology and Critical Care Medicine, Hue Central Hospital, Hue, Vietnam; ^4^Department of Cardiology, 108 Military Central Hospital, Hanoi, Vietnam

## Abstract

**Background:**

Left ventricular systolic dysfunction (LVSD) is common in sepsis. Speckle-tracking echocardiography (STE) is a useful emerging tool for evaluating the intrinsic left ventricular systolic function. High-sensitivity cardiac troponin T (hs-cTnT) is the most sensitive biomarker of myocardial injury. However, there are limited data regarding the association between hs-cTnT level and left ventricular systolic dysfunction based on STE in septic patients. We performed this prospective study to evaluate the diagnostic value of hs-cTnT level for subclinical left ventricular systolic dysfunction measured by STE in septic patients according to the sepsis-3 definition.

**Methods:**

Patients with sepsis based on sepsis-3 definition admitted to the intensive care unit were prospectively performed STE and hs-cTnT level within 24 hours after the onset of sepsis. Baseline clinical and echocardiographic variables were collected. Left ventricular systolic dysfunction was defined as a global longitudinal strain of ≥−15%.

**Results:**

During a 19-month period, 116 patients were enrolled in the study. The elevated hs-cTnT level was seen in 86.2% of septic patients, and 43.1% of patients had LVSD on STE. The median hs-cTnT level and the proportion of elevated hs-cTnT level (>14 ng/L) were significantly higher in patients with LVSD than in patients without LVSD. The area under the ROC curves of hs-cTnT to detect LVSD was 0.73 (*P* < 0.001). In the multivariate analysis, hs-cTnT (HR, 1.002; 95% CI, 1.000 to 1.004; *P* = 0.025) and septic shock (HR, 7.6; 95% CI, 2.25 to 25.76; *P* = 0.001) were independent predictors of LVSD.

**Conclusion:**

Our study indicated that the serum hs-cTnT level might be a useful biomarker for detecting LVSD in septic patients.

## 1. Introduction

Sepsis is one of the leading causes of mortality in the intensive care unit, as it is often associated with multiorgan failure [1, 2]. The heart is a commonly affected organ in sepsis. Approximately 50% of septic patients show signs of myocardial dysfunction [3]. Cardiac abnormalities exhibit in several ways, such as myocardial injury with elevated cardiac biomarker, myocardial dysfunction on echocardiography, and hemodynamic instability [4]. Sepsis-induced myocardial dysfunction (SIMD) can involve both the left and right ventricles and may include both systolic and diastolic dysfunctions [5, 6]. SIMD is related to a significantly high mortality rate of 70–90% [5, 6].

Echocardiography has been a golden tool for evaluating sepsis-induced myocardial dysfunction [7]. However, using the left ventricular ejection fraction (LVEF) to evaluate left ventricular (LV) systolic function measured by conventional echocardiography depends on preload and afterload in septic patients [8–11]. Speckle-tracking echocardiography (STE) is an emerging tool for assessing intrinsic left ventricular function. Left ventricular global longitudinal strain (GLS) measured by speckle-tracking echocardiography is more reliable, reproducible, and sensitive than LVEF in detecting systolic myocardial dysfunction [12, 13].

High-sensitivity cardiac troponin T (hs-cTnT) is the sensitive biomarker of myocardial damage. It is related to prognosis in several conditions such as renal failure, heart failure, pulmonary embolism, trauma, and stroke [14, 15]. Previous studies have shown that an elevated hs-cTnT level may occur in up to 60% of septic patients and is associated with poor outcomes, including increased mortality and longer length of stay in an intensive care unit [16, 17].

Mechanisms of elevated troponin T in sepsis include inflammation, increased myocardial wall stress due to volume overload, toxicity by medications, and renal dysfunction [18]. The elevations of cardiac troponin T correlate with the presence of LV systolic dysfunction on echocardiography [17, 19]. However, data are limited regarding the relationship between hs-cTnT elevation and LV systolic dysfunction measured by speckle-tracking echocardiography in septic patients.

Thus, the purpose of this study was to evaluate the diagnostic value of serum hs-cTnT level for the detection of subclinical left ventricular systolic dysfunction (LVSD) in septic patients. We hypothesized that the elevated hs-cTnT level is associated with LVSD by speckle-tracking echocardiography in patients with sepsis.

## 2. Materials and Methods

We conducted a subanalysis of data that were collected previously in an observational study with the purpose to evaluate the left ventricular systolic dysfunction using STE in patients with septic shock [20].

### 2.1. Study Population

As reported previously [20], between May 2017 and December 2018, we conducted a single-center, cross-sectional study including consecutive patients (≥18 years old) admitted for sepsis or septic shock according to the sepsis-3 definition [21]. Patients with suspected bacterial infection combined with a SOFA score of 2 points or more from the baseline were diagnosed with sepsis [21]. Septic shock was defined as sepsis with persisting hypotension requiring vasopressors to keep a mean arterial pressure (MAP) of ≥65 mmHg and a serum lactate level of ≥2 mmol/L despite adequate fluid resuscitation [21].

Patients were excluded from the study if they had evidence of coronary artery disease, heart failure, moderate to severe valvular heart disease, cardiac valve replacement surgery, cardiac arrhythmia, postcardiac arrest syndrome, low-quality echocardiographic image, and patients or their relatives declined participation. We also excluded any patients without the serum level of hs-cTnT within 24 h of diagnosis of sepsis or septic shock.

Baseline clinical variables including age, gender, comorbidities, hemodynamic parameters, routine blood test results, SOFA score [22] and APACHE II score [23], and speckle-tracking echocardiographic data were collected within 24 h of diagnosis of sepsis or septic shock.

The research protocol was approved by the Ethical Committee of 108 Military Central Hospital. Written informed consent was obtained from the patients or their legal representatives.

### 2.2. High-Sensitivity Cardiac Troponin T Measurements

The serum level of hs-cTnT had been checked at the same time as echocardiography exams in all the patients within the first 24 hours of sepsis or septic shock. Blood samples were processed immediately after they were obtained at the Department of Biochemistry of 108 Military Central Hospital. Measurements of hs-cTnT were conducted on an autoanalyzer (Cobas e 601) using only commercial assays (Roche Diagnostics). The elevated hs-cTnT level was considered if it was >14 ng/L [24].

### 2.3. Two-Dimensional Speckle-Tracking Echocardiography

Two-dimensional speckle-tracking echocardiography was performed using a commercially available echocardiography machine (Vivid S5; GE Healthcare, USA). All echocardiograms were conducted by cardiologists according to the American Society of Echocardiography guidelines [25].

Echocardiographic cine loops were collected by recording a minimum of three consecutive cardiac cycles. Images were collected at a frame rate from 50 to 90 frames/s and digitally transferred to dedicated software for offline analysis. Speckle-tracking echocardiography analysis was conducted for each patient using offline software with the EchoPAC workstation (version 112; GE Healthcare, USA).

Left ventricular global longitudinal strain was calculated by the mean peak systolic values of the 18 segments across three standard apical views (three-chamber, four-chamber, and two-chamber long-axis views) [25, 26]. The software automatically creates a region of interest (ROI), including the entire width of the myocardium, by using a point-and-click approach. After manual adjustment of the width and shape of ROI, the software automatically divides the ROI into six segments, and six corresponding time-strain curves were generated [26]. Peak systolic longitudinal strain values were measured in each apical view. GLS is presented as a percent change (%). Less negative GLS or reduced absolute GLS values show diminished myocardial contractile function. Subclinical left ventricular systolic dysfunction was defined by a GLS of ≥−15% (less negative than −15%) according to the previous studies [27–29].

### 2.4. Statistical Analysis

SPSS, version 20.0, statistical software (SPSS, Inc., Chicago, IL, USA) was used for statistical analysis. Continuous variables were presented as mean values and standard deviation (SD) or median (interquartile range). Categorical variables were expressed as frequencies and percentages. Continuous variables were compared using the Student *t*-test or the MannWhitney test. Comparisons were performed using the chi-squared test or the Fisher exact test for categorical variables, as appropriate. Septic patients were categorized into one of 2 groups according to LV systolic function measured by speckle-tracking echocardiography (GLS≥−15% and GLS<−15%).

A receiver operating characteristic (ROC) curve was used to detect the cutoff value of hs-cTnT in predicting subclinical LV systolic dysfunction. The best cutoff point was selected as the maximum value of the sum of sensitivity and specificity [30]. Comparisons of each predictor were performed using MedCalc 18.2 software (Acacialaan, Ostend, Belgium). Univariate regression analyses were performed to find the factors related to subclinical LVSD. The variables with *P* value <0.05 on the univariate analysis were entered, and the collinearity was evaluated before being included in the multivariate regression model to further confirm the independent predictor of LVSD. Data are presented as odds ratios with the corresponding 95% confidence intervals. Two-tailed *P* values < 0.05 were considered statistically significant.

## 3. Results

### 3.1. Characteristics of the Study Subjects

A total of 127 patients were included in the original study [20]. Eleven were excluded from analysis due to a lack of hs-cTnT level measurements. The remaining 116 patients were eligible for assessment.

The baseline clinical and laboratory characteristics of the patients are presented in Table 1.

There were 78.4% of male, with the mean age of 67.3 ± 15.9 years, mean SOFA score of 8.9, and mean APACHE II score of 19.1. Thirty-three patients (28.5%) had sepsis, and the remaining 83 patients (71.5%) had septic shock. Ninety patients (79.3%) received mechanical ventilation, and 53 patients (45.7%) received continuous renal replacement therapy. The in-hospital mortality rate was 34.5%.

There were no significant differences in gender, age, comorbidities, and mean length of stay in the ICU between the patients with LVSD and those without LVSD (*P* > 0.05). APACHE II and SOFA scores were significantly higher in patients with LV systolic dysfunction than those with normal LV function (*P* < 0.05). The proportion of patients with LVSD receiving mechanical ventilation and continuous renal replacement therapy was higher than patients without LVSD (*P* < 0.05).

There was no significant difference in inflammatory markers, including procalcitonin (PCT) (*P* = 0.440) and white blood cell (WBC) (*P* = 0.994) between the two groups. The median hs-cTnT level and the proportion of hs-cTnT elevation (>14 ng/L) were significantly higher in the group of patients with LVSD than in the group of patients without LVSD.

Heart rate and central venous pressure (CVP) were not significantly different between the two groups. Mean arterial pressure was significantly lower in patients with LVSD (*P* < 0.001). The proportion of septic shock in a group of patients with LVSD was higher than that in a group of patients without LVSD (92% versus 56%, *P* < 0.001).

### 3.2. Echocardiographic Variables

Table 1 summarizes the echocardiographic parameters of the subjects. The patients with LVSD had less negative GLS than patients without LVSD (*P* < 0.001).

### 3.3. Diagnostic Value of High-Sensitive Cardiac Troponin T

**Figure 1:**
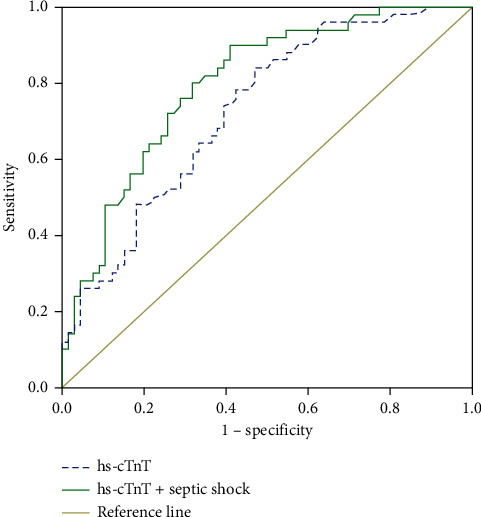
Receiver operating characteristic (ROC) curves for hs-cTnT and hs-cTnT combined with septic shock to predict subclinical LV systolic dysfunction. The serum hs-cTnT level had power for predicting subclinical LV systolic dysfunction as suggested by the area under the curve (AUC) of 0.73, *P* < 0.001.

The ROC analysis results for serum hs-cTnT levels to detect subclinical LV systolic dysfunction are shown in Figure 1. The area under the curve was 0.73. The serum hs-cTnT level of ≥40 ng/L was proposed as the optimal cutoff value, which provided a sensitivity of 84% and a specificity of 53% for the prediction of subclinical LV systolic dysfunction in sepsis.

The AUC of a combination of hs-cTnT and septic shock was significantly higher than that of hs-cTnT level alone in predicting LVSD in sepsis (0.80 versus 0.73, *P* = 0.015). The combination model sensitivity and specificity were 90% and 59.1%, respectively. The detailed results are shown in Table 2.

### 3.4. Logistic Regression Analysis

Univariate analysis using the logistic regression model was performed to examine the associations of each variable with subclinical LV systolic dysfunction. The results showed that the septic shock, mechanical ventilation, and hs-cTnT level were associated with the risk of LVSD (Table 3). In the multivariate analysis, the hs-cTnT level remained the independent predictor of LVSD in sepsis (HR, 1.002; 95% CI, 1.000 to 1.004; *P* = 0.025). Moreover, the septic shock was also an independent predictor (HR, 7.6; 95% CI, 2.25 to 25.76; *P* = 0.001).

## 4. Discussion

In this study, we showed that the elevated hs-cTnT level was seen in 86.2% of septic patients, and 43.1% of patients showed subclinical LVSD on speckle-tracking echocardiography. The hs-cTnT level and the percentage of elevated hs-cTnT level were significantly greater in patients with LVSD than in patients without LVSD. The serum level of hs-cTnT is the most sensitive and specific marker of cardiomyocyte necrosis, which is widely used in the diagnosis of patients with acute coronary syndromes [31, 32]. Moreover, the elevation of cardiac troponin has also been seen in other cardiac and noncardiac conditions, including heart failure [33], pulmonary embolism [34], and kidney failure [35]. Some previous studies demonstrated that the serum level of hs-cTnT increases significantly in septic patients [16, 17].

The underlying mechanism of elevated troponin in sepsis has not been clarified yet. Elevations of cardiac troponin may correlate with the presence of left ventricular systolic dysfunction based on echocardiography and pulmonary artery catheterization [17, 19, 36]. Furthermore, several studies have shown that reduced coronary blood flow secondary to arterial hypotension, myocardial depressant factors, nitric oxide, proinflammatory cytokines, and mitochondrial dysfunction play important roles in the mechanism of cardiac dysfunction [6]. Changes in the cell membrane's permeability to ion channels may also contribute to troponin release from the intracellular space [37].

Approximately 50% of septic patients may develop ventricular function impairment, referred to as sepsis-induced myocardial dysfunction [37]. Echocardiography is a widely accepted standard for evaluating changes in LV function and helping to identify SIMD. Conventional echocardiography based on LV ejection fraction (LVEF) is dependent on LV loading conditions, especially preload and afterload changes [38]. Speckle-tracking echocardiography is a novel imaging technique and recently applied in clinical practice to assess intrinsic LV systolic function in septic patients to resolve several limitations of LVEF [39–42]. However, limitations of echocardiography are not easily measured, time-consuming, and difficult to perform in septic patients due to postural influence and mechanical ventilation. Thus, simple bedside methods are necessary to access cardiac function. The hs-cTnT is an easy, cost-effective, reliable test to identify cardiac dysfunction.

In this study, we assessed the association between hs-cTnT levels and LV systolic dysfunction measured by speckle-tracking echocardiography in septic patients according to the sepsis-3 definition. We found that the hs-cTnT level has a fairly good diagnostic value for detecting LV systolic dysfunction. The combination of hs-cTnT and septic shock improved the predictive value of hs-cTnT alone (0.80 versus 0.73, *P* = 0.015). In the multivariate analysis, the hs-cTnT level was the independent predictor of LVSD in septic patients. Similar results were found by Kim et al., the AUC of hs-cTnI in predicting SIMD was 0.668, and an elevated hs-cTnI level (≥40 ng/L) at admission showed SIMD with the sensitivity (58.6%) and specificity (59.1%) [19]. The hs-cTnI level was associated with SIMD (OR 1.03; 95% CI 1.01 to 1.06; *P* = 0.008) [19]. Landesberg et al. demonstrated that the hs-cTnT level correlated with LV diastolic dysfunction and RV dilatation in patients with severe sepsis and septic shock [43]. Ver Elst et al. also reported that both cTnI and cTnT were associated with LV dysfunction measured by two-dimensional transesophageal echocardiography in septic patients (*P* < 0.0001) [17]. Ammann et al. showed that the elevated troponin level was associated with decreased left ventricular systolic function based on left ventricular ejection fraction (LVEF) [44].

To the best of our knowledge, this is the first prospective study evaluating the performance of hs-cTnT for discrimination of LV systolic dysfunction based on speckle-tracking echocardiography in septic patients.

### 4.1. Limitations

There were several limitations to the present study. First, this was a single-center study with a small sample size, so the larger prospective studies with a more meticulous design are needed. Second, LV systolic function assessment was performed only at admission, so it did not show an association between improvement/decline in LV systolic function with changes in serum hs-cTnT levels. Third, LV diastolic function was not evaluated.

## 5. Conclusion

Our study has shown that the hs-cTnT level is associated with left ventricular systolic dysfunction based on speckle-tracking echocardiography. The serum hs-cTnT level has a fairly good diagnostic potential for the identification of left ventricular systolic dysfunction in septic patients. However, further studies involving more patients are necessary to validate the results of this study.

## Figures and Tables

**Table 1:** Baseline characteristics, biochemical, hemodynamic, and echocardiographic data of patients classified in relation to the presence of left ventricular systolic dysfunction.

	All patients (*n* = 116)	Patients without LVSD *(n* = 66)	Patients with LVSD (*n* = 50)	*P* value
*Characteristics*				
Age, mean (years)	67.3 ± 15.9	65.9 ± 15.9	69.1 ± 16.0	0.280
Male, *n* (%)	91 (78.4)	54 (81.8)	37 (74.0)	0.311
Heart rate (bpm)	103.6 ± 19.3	101.3 ± 19.8	106.8 ± 18.3	0.139
MAP (mmHg)	78.0 ± 15.0	82.2 ± 14.8	72.3 ± 13.5	<0.001^*∗*^
CVP (mmHg)	7.0 ± 2.6	6.8 ± 2.3	7.4 ± 2.9	0.261
SOFA score	8.9 ± 4.1	7.3 ± 3.8	11.0 ± 3.6	<0.001^*∗*^
APACHE II score	18.1 ± 8.1	14.7 ± 6.9	22.6 ± 7.4	<0.001^*∗*^
Shock, *n* (%)	83 (71.5)	37 (56.0)	46 (92.0)	<0.001^*∗*^
CRRT, *n* (%)	53 (45.7)	20 (30.3)	33 (66.0)	<0.001^*∗*^
LOS in ICU (days)	7.1 ± 6.3	6.5 ± 5.4	7.8 ± 7.4	0.289
Mechanical ventilation, *n* (%)	92 (79.3)	48 (72.7)	44 (88.0)	0.044^*∗*^
In-hospital mortality, *n* (%)	40 (34.5)	10 (14.1)	30 (53.6)	<0.001^*∗*^

*Comorbidities*				
Hypertension, *n* (%)	50 (43.1)	27 (40.9)	23 (46.0)	0.583
Type 2 DM, *n* (%)	25 (21.5)	11 (16.7)	14 (28.0)	0.142
Stroke, *n* (%)	14 (12.1)	10 (15.1)	4 (8.0)	0.242
CKD, *n* (%)	21 (18.1)	8 (12.1)	13 (23.6)	0.055
COPD, *n* (%)	5 (4.3)	3 (4.5)	2 (4.0)	0.886

*Laboratory data*				
WBC (×10^9^/L)	14.6 ± 10.1	14.6 ± 10.0	15.5 ± 10.5	0.994
Lactate (mmol/L)	3.4 (2.0–6.9)	2.7 (1.8–4.7)	5.9 (2.6–8.8)	<0.001^*∗*^
Procalcitonin (ng/mL)	31.5 (6.4–98.8)	16.2 (3.6–82.5)	46.1 (15.0–100.0)	0.440
hs-cTnT (ng/L)	55 (30–153)	38 (19–109)	113 (48–334)	0.005^*∗*^
hs-cTnT > 14 ng/L, *n* (%)	100 (86.2)	52 (78.8)	48 (96.0)	0.007^*∗*^

*Speckle-tracking echocardiographic*				
LS-A4C (%)	−15.4 ± 3.3	−17.4 ± 2.2	−12.7 ± 2.5	<0.001^*∗*^
LS-A2C (%)	−15.2 ± 3.8	−17.5 ± 2.5	−12.1 ± 3.0	<0.001^*∗*^
LS-A3C (%)	−15.8 ± 3.5	−17.9 ± 2.3	−13.1 ± 2.7	<0.001^*∗*^
GLS (%)	−15.5 ± 3.2	−17.6 ± 1.8	−12.6 ± 2.4	<0.001^*∗*^

*Note*. Continuous data are presented as means ± SD or median (interquartile range) and categorical data as *n* (%). A3C, apical three-chamber view; A4C, apical four-chamber view; A2C, apical two-chamber view; APACHE II, acute physiology and chronic health evaluation; COPD, chronic obstructive pulmonary disease; CKD, chronic kidney disease; CRRT, continuous renal replacement therapy; CVP, central venous pressure; DM, diabetes mellitus; GLS, global longitudinal strain by speckle-tracking echocardiography; hs-cTnT, high-sensitivity cardiac troponin T; LS, longitudinal strain; ICU, intensive care unit; LOS, length of stay; MAP, mean arterial pressure; SOFA, sequential organ failure assessment; WBC, white blood cell; ^*∗*^*P* < 0.05.

**Table 2:** Performance of variables in predicting left ventricular systolic dysfunction.

Variables	AUC ROC	*P* value	Cutoff value	Sensitivity (%)	Specificity (%)
hs-cTnT (ng/L)	0.73	<0.001	>40	84	53
hs-cTnT + septic shock	0.80	<0.001		90	59.1

AUC ROC, area under the receiver operating characteristic curve; hs-cTnT, high-sensitivity cardiac troponin T.

**Table 3:** Univariate and multivariate logistic regression analysis for the detection of subclinical left ventricular systolic dysfunction.

Dependent variables	Univariable		Multivariable	
	Hazard ratio (95% CI)	*P*	Hazard ratio (95% CI)	*P* value
Age	1.01 (0.99–1.03)	0.28	—	—
Male gender	0.63 (0.26–1.54)	0.31	—	—
Hypertension	1.23 (0.58–2.58)	0.38	—	—
Type 2 DM	1.90 (0.79–4.75)	0.14	—	—
CKD	2.54 (0.96–6.73)	0.06	—	—
COPD	0.87 (0.14–5.44)	0.88	—	—
Mechanical ventilation	2.75 (1.00–7.55)	0.05	1.70 (0.51–5.73)	0.39
Heart rate	1.01 (0.99–1.03)	0.14	—	—
CVP	1.08 (0.94–1.25)	0.26	—	—
Shock	9.01 (2.90–27.94)	<0.001	7.62 (2.25–25.76)	0.001
hs-cTnT	1.002 (1.000–1.004)	0.026	1.002 (1.000–1.004)	0.025

*Note*. Data are expressed as hazard ratio and 95% confidence interval (CI). SOFA, sequential organ failure assessment; CI, confidence interval; GLS, global longitudinal strain; CKD, chronic kidney disease; COPD, chronic obstructive pulmonary disease; CVP, central venous pressure*;* DM, diabetes mellitus; hs-cTnT, high-sensitivity cardiac troponin T.

## Data Availability

The data used for the findings of this study are available from the corresponding author upon request.

## Abbreviations

APACHE: Acute Physiology and Chronic Health Evaluation
A3C: Apical 3-chamber view
GLS: Global longitudinal strain
A4C: Apical 4-chamber view
A2C: Apical 2-chamber view
AUC: Area under the curve
COPD: Chronic obstructive pulmonary disease
CRRT: Continuous renal replacement therapy
CI: Confidence interval
CKD: Chronic kidney disease
CVP: Central venous pressure
DM: Diabetes mellitus
ICU: Intensive care unit
LV: Left ventricular
HR: Heart rate
hs-cTnT: High-sensitivity cardiac troponin T
SD: Standard deviation
SIMD: Sepsis-induced myocardial dysfunction
SOFA: Sequential organ failure assessment
STE: Speckle-tracking echocardiography
LS: Longitudinal strain
LVSD: Left ventricular systolic dysfunction
ROC: Receiver operating characteristic
OR: Odds ratio
WBC: White blood cell
MAP: Mean arterial pressure
LVEF: Left ventricular ejection fraction.
